# Development of a prognostic index and screening of prognosis related genes based on an immunogenomic landscape analysis of bladder cancer

**DOI:** 10.18632/aging.202917

**Published:** 2021-04-22

**Authors:** GenYi Qu, Zhengsheng Liu, Guang Yang, Yong Xu, Maolin Xiang, Cheng Tang

**Affiliations:** 1 Department of Urology, Zhuzhou Central Hospital, Zhuzhou 412007, China; 2 Department of Urology, The First Affiliated Hospital of Xiamen University, Xiamen 361003, China

**Keywords:** bladder cancer, immune-related genes, immunogenomic landscape, prognostic index, The Cancer Genome Atlas

## Abstract

Background: Bladder cancer (BLCA) is one of the most common urinary tract malignant tumors. It is associated with poor outcomes, and its etiology and pathogenesis are not fully understood. There is great hope for immunotherapy in treating many malignant tumors; therefore, it is worthwhile to explore the use of immunotherapy for BLCA.Methods: Gene expression profiles and clinical information were obtained from The Cancer Genome Atlas (TCGA), and immune-related genes (IRGs) were downloaded from the Immunology Database and Analysis Portal. Differentially-expressed and survival-associated IRGs in patients with BLCA were identified using computational algorithms and Cox regression analysis. We also performed functional enrichment analysis. Based on IRGs, we employed multivariate Cox analysis to develop a new prognostic index.Results: We identified 261 IRGs that were differentially expressed between BLCA tissue and adjacent tissue, 30 of which were significantly associated with the overall survival (all P<0.01). According to multivariate Cox analysis, nine survival-related IRGs (MMP9, PDGFRA, AHNAK, OAS1, OLR1, RAC3, IGF1, PGF, and SH3BP2) were high-risk genes. We developed a prognostic index based on these IRGs and found it accurately predicted BLCA outcomes associated with the TNM stage. Intriguingly, the IRG-based prognostic index reflected infiltration of macrophages.Conclusions: An independent IRG-based prognostic index provides a practical approach for assessing patients' immune status and prognosis with BLCA. This index independently predicted outcomes of BLCA.

## INTRODUCTION

Bladder cancer is the most common malignant tumor of the urinary system. It is the ninth most incident neoplasm in China and the 10th most common malignant tumor worldwide [1, 2]. Bladder cancer incidence increases with age, with the age of peak incidence at 50–70 years. Its incidence in men is 3–4 times greater than that of women [3]. With the aging of the population, changes in living habits, and improvements in diagnostic technology, bladder cancer incidence has increased yearly. Although the diagnosis and treatment of bladder cancer have greatly improved, advanced bladder cancer outcomes remain poor; the 5-year survival rate is low. Although studies have reported some prognostic biomarkers for bladder cancer [4], their utility is reduced by various factors, and the predictive power of individual indicators is insufficient. By contrast, genetic testing provides better predictive performance, and multigene prognostic models guide clinicians in choosing appropriate treatments [5].

The rise of immunotherapy, especially immune checkpoint inhibitors, has changed the treatment mode for advanced bladder cancer [6]; however, its remission rate remains substantial; therefore, it would be helpful to generate an immune-related gene model to stratify the risk of bladder cancer both to predict outcomes and to track treatment. And the study of the immune gene-related model has been reported in a variety of tumors, including colorectal cancer, head and neck cancer, and papillary thyroid cancer [5, 7, 8].

In the present study, based on TCGA, we aimed to identify reliable immune gene-related biomarkers to predict bladder cancer outcomes. We used R software to identify differentially expressed immune genes combined with clinical data from TCGA. We selected immune genes significantly related to outcomes to construct a model that predicts bladder cancer outcomes. Our findings may lay the foundation for in-depth immune-related work and may enable personalized treatment of bladder cancer.

## MATERIALS AND METHODS

### Clinical samples and data collection

Transcriptome RNA-sequencing data and corresponding clinical data of all bladder cancer samples (including 410 bladder cancer samples and 19 non-tumor samples) were downloaded from TCGA (https://cancergenome.nih.gov/), excluding patients with overall survival of <90 days and unclear clinical stage [9]. Each tumor sample corresponded to one patient. The data collection date was June 1, 2020. The list of immune-related genes (IRGs) was downloaded from the immunology database and the analysis portal (ImmPort) database (https://www.immport.org/home) [10]. ImmPort provides accurate and timely immunological data, including IRGs for cancer research. The data shared through ImmPort provides a strong foundation for immunology research. The IRGs we downloaded from this website were involved in immune activity [11].

### Analysis of differentially expressed genes

Transcriptome RNA-sequencing data was collated and standardized. Differential gene analysis returned a list of significantly differentially expressed genes (DEGs) using the limma package in R software [12], with the log2 | fold change | >1 and the false discovery rate <0.05 as the cutoff values [8]. We created heat maps of DEGs using the pheatmap package and drew differential gene expression volcano plots using the ggplot2 package [13, 14]. Then, we extracted differentially expressed IRGs from the intersection of immune genes and all DEGs.

### Differential immune gene analysis

The search tool STRING (https://string-db.org/) allows searches for interacting genes; it is a biologically predictive web resource containing many proteins and known interaction functions [15]. We used the correlations of these functions and expression levels to analyze and evaluate the interactions of DEGs. We designated a composite score greater than 0.4 as the cutoff criterion. Based on STRING information, we built a PPI network using Cytoscape software [16] (version 3.7.2).

### Survival-related IRGs and survival analysis

We downloaded the clinical characteristics and follow-up data from TCGA and selected overall survival (OS) as the primary endpoint; we then analyzed and sorted the data using Perl software. We used a univariate Cox regression analysis to select genes related to survival (false discovery rate <0.05). Based on the Schoenfeld residual (phtest) of the Cox regression model, we made proportional hazards assumptions. The significance value of the overall proportional hazard test was less than 0.01 (P <0.01). The hazard ratio (HR) is the ratio of the expression of IRGs between tumor samples and standard samples. We defined high-risk IRG (HR >1) and low-risk IRG (HR <1), with HR = 1 as the critical value.

### Transcription factor-mediated regulatory network

Transcription factors (TFs) control gene expression, including IRG, and play a vital role in regulating immune function. Therefore, it is necessary to explore the interaction between survival-related IRGs and TFs. First, we downloaded 318 TFs from the Cistrome Cancer database (http://cistrome.org/CistromeCancer/) [17]. Then, we extracted differentially expressed TFs from all DEGs to draw expression heat maps and volcano maps. Subsequently, we used R software to carry out correlation analysis of differentially expressed TFs and survival-related IRGs. If the |correlation value| was >0.6 and P <0.05, the correlation was reliable. We constructed a TF-mediated regulatory network for high-risk survival-related IRGs (HR >1) and potential TF using Cytoscape software.

### Development of the IRG-based prognostic index

We used expression data and coefficients of these survival-related IRGs to develop an IRG-based prognostic index (IRGPI) using multivariate analysis. We used multivariate Cox regression analysis to calculate the correlations between risk scores and OS and identify potential prognostic IRGs, with integrated IRGs remaining independent prognostic indicators. Specifically, we constructed the IRGPI by multiplying the expression value with the Cox regression coefficient [18].

### Assessment of IRGPI and genetic alteration analysis

We classified patients as high-risk or low-risk based on IRGPI values and used the R package pheatmap to draw risk curves. We drew corresponding Kaplan–Meier survival curves to show the OS of the various risk groups. We drew receiver operating characteristic (ROC) curves to assess the sensitivity and specificity of the model. IRGPI and clinicopathological factors were analyzed for single factor and multi-factor survival. These analyses were performed using the R package survival [19]. We also explored the correlation between hub IRG expression and clinicopathological factors.

### Immune cell correlation analysis

The Tumor Immune Estimation Resource (TIMER https://cistrome.shinyapps.io/timer/) is an online database that contains tumor-infiltrating immune cells. We obtained infiltration levels of six immune cells (B cells, CD4+ T cells, CD8+ T cells, neutrophils, macrophages, and dendritic cells) from TCGA and other public validation databases containing tumor information. TIMER reanalyzed gene expression data. Using the database, we determined the abundance of tumor-infiltrating immune cells of six subtypes and the relationships between immune cell infiltration and other parameters. Studies confirmed that the platform is appropriate for the present study [8, 20, 21]. We downloaded immune infiltration levels of patients with BLCA from TIMER and calculated the correlations between IRGPI and immune cell infiltration. P <0.05 was considered statistically significant.

### Statistical analysis

We used R software (version 3.6.1) and R-associated packages to perform function enrichment analysis, differential analysis of immune genes, Cox regression analysis, and survival analysis [22]. We used survival and survminer packages in R to create Kaplan–Meier curves and survival ROC curves. We used these findings to assess the performance of IRGPI based on the area under the curve (AUC) of the survival ROC curve [23]. We used an independent *t*-test to calculate the differences between clinical features and prognosis-related IRGs. P <0.05 was considered statistically significant.

## RESULTS

### Identification of differentially expressed IRGs

**Figure 1:**
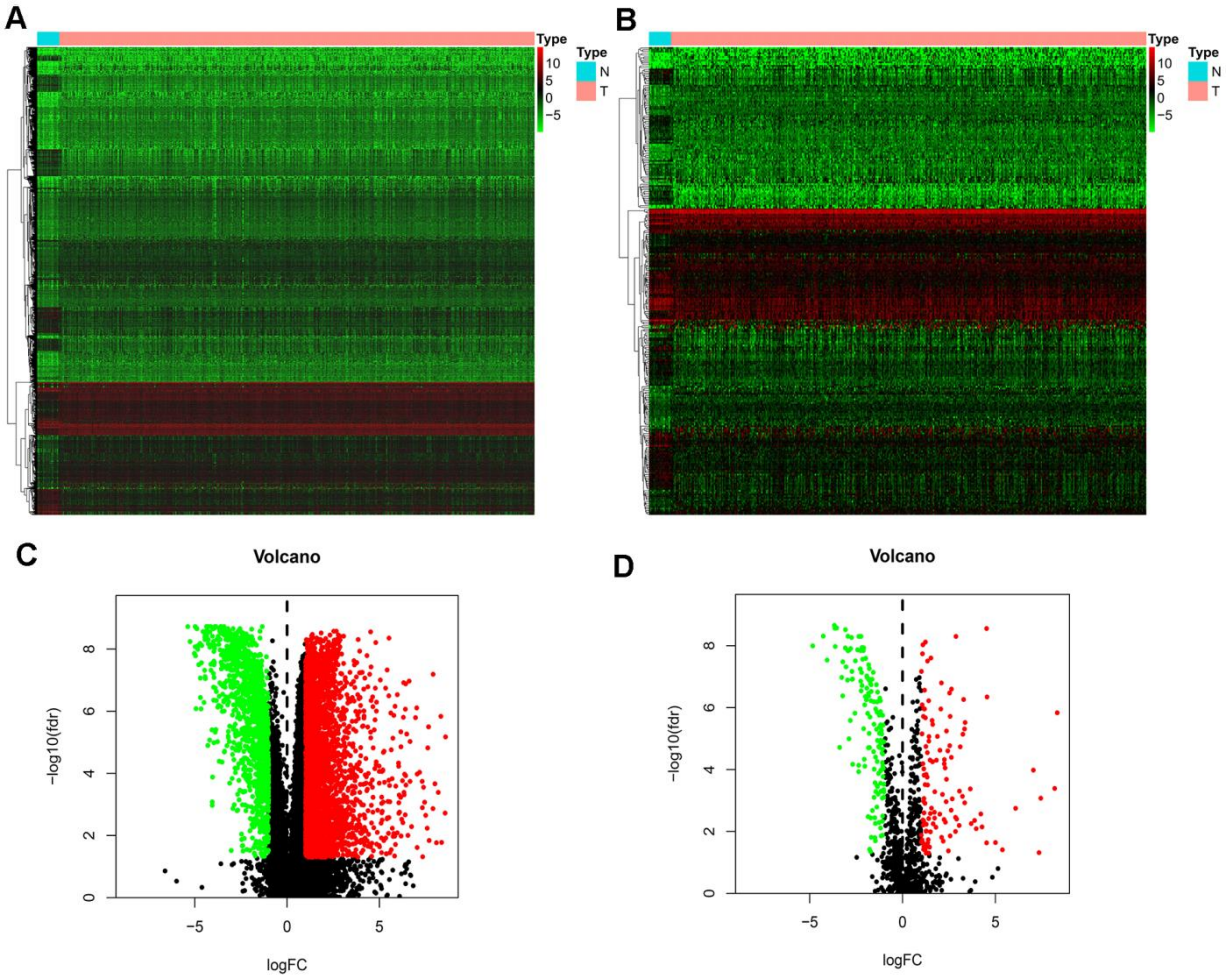
**Differentially expressed IRGs.** (**A**) Heatmap demonstrating DEGs between BLCA and normal samples, with red representing high expression and green representing low expression. (**B**) Heatmap demonstrating differentially expressed IRGs between BLCA and normal samples, with red representing high expression and green representing low expression. (**C**) Volcano plot of 4893 DEGs, with red representing up-regulated DEGs and green representing down-regulated DEGs. (**D**) volcano plot of 261 differentially expressed IRGs, with red representing up-regulated IRGs and green representing down-regulated IRGs.

We downloaded 410 BLCA samples and 19 normal samples, including a total of 18769 genes. We identified 4893 DEGs using the R limma package; these included 3468 up-regulated DEGs and 1425 down-regulated DEGs (Figure 1A, 1C). Using the list of IRGs, we identified 261 differentially expressed IRGs, including 120 up-regulated, differentially expressed IRGs, and 141 down-regulated, differentially expressed IRGs (Figure 1B, 1D).

### Identification of survival-associated IRGs

**Figure 2:**
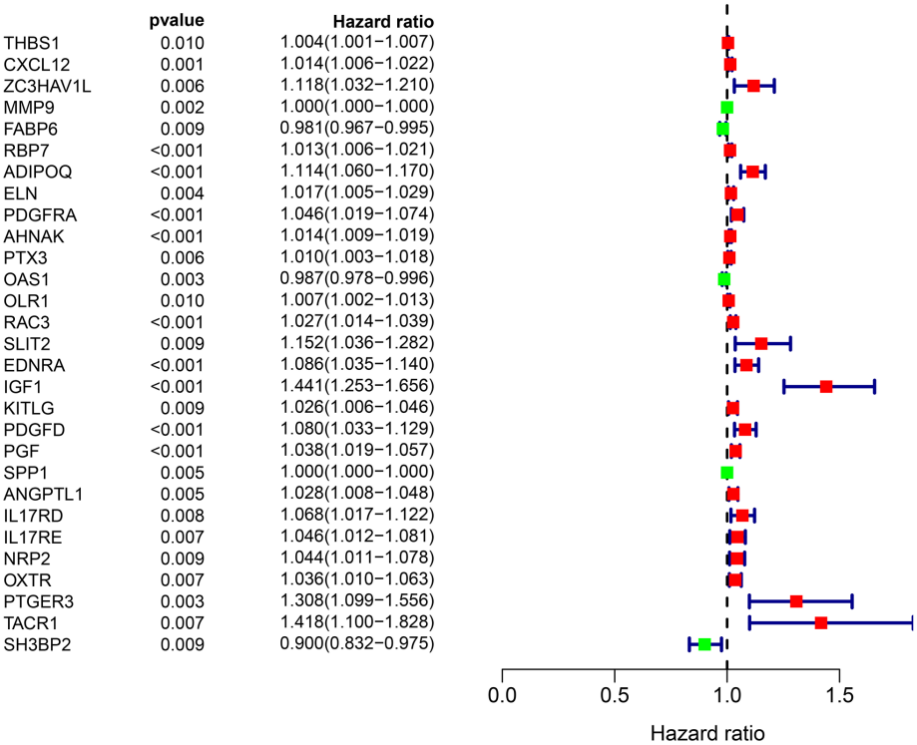
Forest plot of the hazard ratios showing the prognostic values of survival-associated IRGs, red dots represent high-risk genes (HR > 1), and green dots represent low-risk genes (HR < 1).

To identity the differentially survival-associated IRGs, we performed univariate Cox regression analysis on the expression of 261 differentially expressed IRGs in BLCA. We found that the expression of 26 differentially survival-associated IRGs significantly correlated with OS in patients with BLCA (all P <0.001) (Table 1). The forest plot results in Figure 2 show the prognostic value of these IRGs in patients with BLCA. There were 23 genes with HR >1 and three genes with HR <1. This indicates that most IRGs are high-risk genes for the outcome of BLCA.

**Table 1:** General characteristics of BLCA-specific immune-related genes.

**Gene symbol**	**LogFC**	**FDR**	**HR**	**P-value**
THBS1	-2.364170784	0.0001167	1.004234339	0.009624386
CXCL12	-1.793919298	5.48E-08	1.013942892	0.001039896
ZC3HAV1L	1.32960684	0.000174748	1.117544502	0.006339535
MMP9	3.04862784	0.006420263	1.000283778	0.001779972
FABP6	2.003815556	0.002768244	0.980771613	0.008948499
RBP7	-1.120977579	7.12E-06	1.013395606	0.000240158
ADIPOQ	-1.228311896	4.98E-07	1.113856355	1.90E-05
ELN	-1.951814757	5.57E-07	1.017100402	0.00424009
PDGFRA	-1.877812889	1.53E-06	1.046033385	0.000892835
AHNAK	-1.013000982	0.006016427	1.013593551	5.82E-08
PTX3	-2.032872149	7.73E-05	1.010441923	0.006173227
OAS1	1.161396793	0.00249904	0.986847766	0.003445374
OLR1	2.847420735	0.009974211	1.007461503	0.009630675
RAC3	2.871069381	4.99E-09	1.026505382	1.66E-05
SLIT2	-2.537542555	3.07E-08	1.152167049	0.009068295
EDNRA	-1.795229834	6.78E-06	1.086359281	0.00075286
IGF1	-1.186085678	3.59E-06	1.440812129	2.84E-07
KITLG	-1.011084991	0.000780218	1.025879879	0.008832397
PDGFD	-1.75479763	1.99E-06	1.07999107	0.000619345
PGF	1.55827545	0.001770518	1.037799866	6.85E-05
SPP1	4.546905253	4.53E-07	1.000160276	0.005339656
ANGPTL1	-2.694001199	1.19E-07	1.028080936	0.004962655
IL17RD	-1.187999641	0.007763757	1.068321126	0.008041726
IL17RE	1.146456501	0.004545266	1.045799441	0.007436093
NRP2	-1.387343245	0.001626737	1.043984621	0.009260416
OXTR	2.293078486	8.88E-05	1.03604654	0.006923307
PTGER3	-1.842483391	1.97E-05	1.307642614	0.002505678
TACR1	-2.18286288	1.15E-08	1.417848098	0.00706276
SH3BP2	1.132467385	1.07E-06	0.900277442	0.009410188

### Transcription factor regulatory network

**Figure 3:**
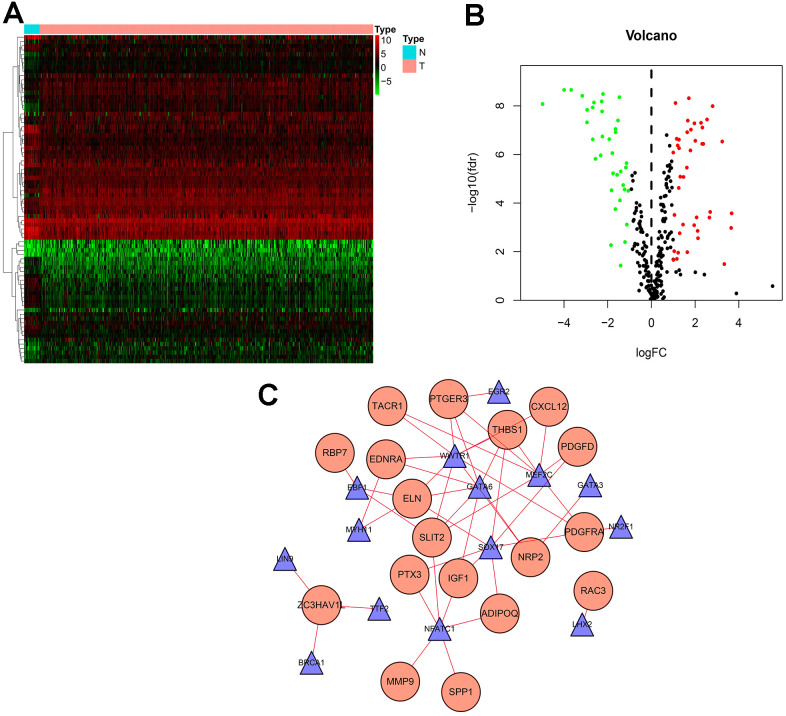
**Transcription factor (TF) regulatory network.** Differentially expressed TFs in the DEGs between BLCA samples and normal samples. (**A**) The heatmap and (**B**) volcano plot of differentially expressed TFs. (**C**) In the-mediated regulatory network, triangles represent TFs; circles represent IRGs.

TF plays a crucial role in regulating molecular networks. To explore the molecular mechanisms between survival-associated IRGs and TF, we downloaded 318 tumor-related TFs from the cancer database to study their regulatory mechanisms. We identified 77 differentially expressed TFs (Figure 3A, 3B) in genes that were differentially expressed between BLCA samples and normal samples, of which 41 were up-regulated and 36 were down-regulated (Figure 3A, 3B). To study the relationship between differentially expressed TF and survival-associated IRGs, we constructed a TF-mediated regulatory network based on 18 TFs and eight survival-associated IRGs (Figure 3C). WWTR1 had the most connections with other survival-associated IRGs, while THBS1 had the most connections with other TFs.

### Development of IRGPI

**Figure 4:**
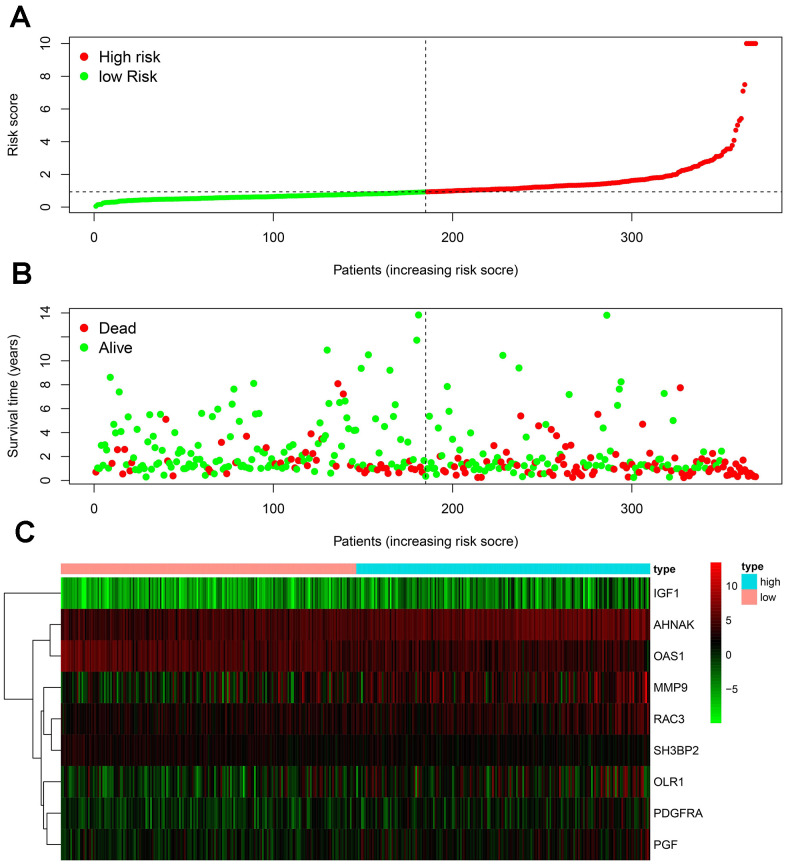
**Development of the IRGPI.** (**A**) Distribution of patients with high-risk scores (red color) and low-risk scores (green color); (**B**) survival status of patients with BLCA (red dots stand for the deceased patients and the green dots stand for the survivors); (**C**) heatmap of the nine survival-associated IRGs expression profiles.

**Figure 5:**
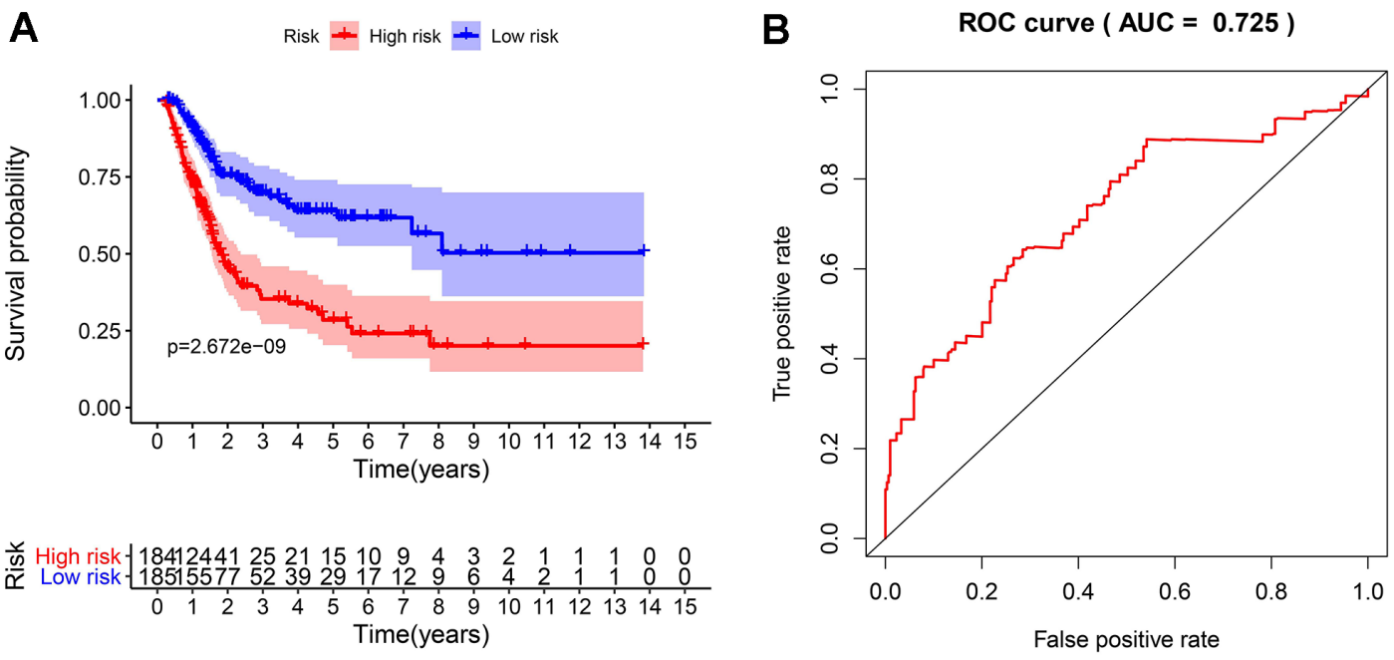
**The evaluation of the IRGPI.** (**A**) The Kaplan-Meier curves of OS for patients with high-risk scores (red line) and low-risk scores (blue line); (**B**) Verification of the accuracy of the IRGPI based on analysis of the AUC of the survival-dependent ROC curve.

**Figure 6:**
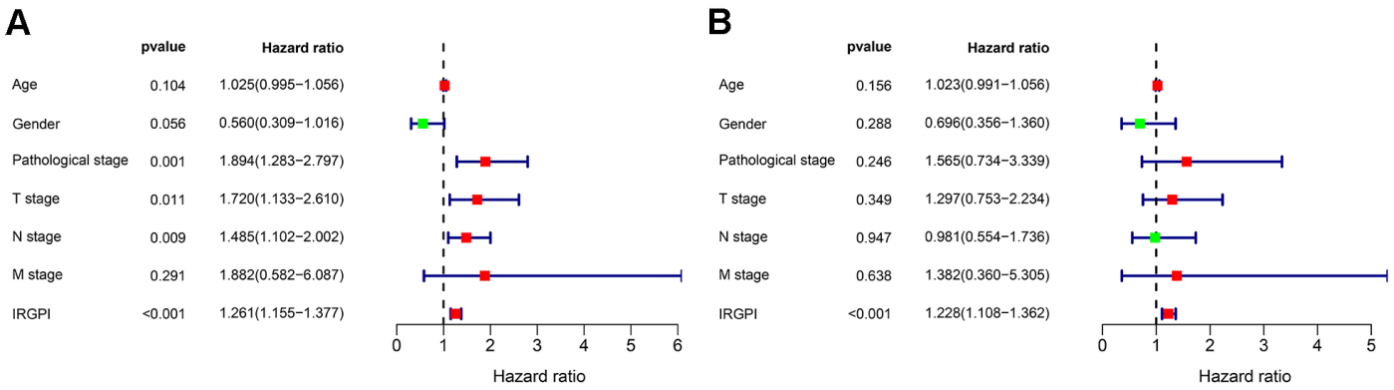
Univariate (**A**) and multivariate (**B**) Cox regression analysis in terms of OS for patients with BLCA.

To develop an IRGPI, we identified nine survival-associated IRGs for BLCA using multivariate Cox regression analysis, and we constructed the optimal IRGPI to group patients with BLCA (Figure 4). We calculated risk scores based on expression levels of nine survival-associated IRGs and regression coefficients, with the following formula: risk score = [expression level of MMP9×(0.0003)] + [expression level of PDGFRA×(0.0290)] + [expression level of AHNAK×(0.0124)] + [expression level of OAS1×(−0.0084)] + [expression level of OLR1×(0.0053)] + [expression level of RAC3×(0.0229) + [expression level of IGF1×(0.2830)] + [expression level of PGF×(0.0180)] + [expression level of SH3BP2×(−0.0788)]. We divided patients with BLCA into a high-risk group (n = 184) and a low-risk group (n = 185) according to the median risk score. The distribution of risk scores and survival status are shown in Figure 4. High-risk patients died more often than did low-risk patients. According to Kaplan–Meier survival analysis, OS was significantly lower in the high-risk group than in the low-risk group (P = 2.672e^−09^) (Figure 5). The five-year survival rate for the high-risk group was 51.63%, while the five-year survival rate for the low-risk group was 25.95% (Figure 5). We generated ROC curves, and the AUC was calculated to evaluate the prediction accuracy of the IRGPI. The area was 0.725, suggesting that the IRGPI has excellent potential for predicting patients' survival with BLCA (Figure 5). Univariate and multivariate analysis (Table 2) suggested that IRGPI significantly correlates with survival in BLCA. The pathological stage, T stage, N stage, and IRGPI were independent predictors (Table 2). However, the multivariate analysis suggested that only IRGPI was an independent predictor of outcome in BLCA after adjustment for all relevant clinical factors (Figure 6).

**Table 2:** Univariate and multivariate Cox regression analysis of BLCA.

**Variables**	**Univariate analysis**			**Multivariate analysis**	
	**HR (95% CI)**	***P* value**		**HR (95% CI)**	***P* value**
Age	1.025(0.995-1.056)	0.104		1.023(0.991-1.056)	0.156
Gender	0.560(0.309-1.016)	0.056		0.696(0.356-1.360)	0.288
Pathological stage	1.894(1.283-2.797)	0.001		1.565(0.734-3.339)	0.246
T stage	1.720(1.133-2.610)	0.011		1.297(0.753-2.234)	0.349
N stage	1.485(1.102-2.002)	0.009		0.981(0.554-1.736)	0.947
M stage	1.881(0.582-6.087)	0.291		1.382(0.360-5.305)	0.638
IRGPI	1.261(1.155-1.377)	<0.001		1.228(1.108-1.362)	<0.001

### Clinical correlation analysis

**Figure 7:**
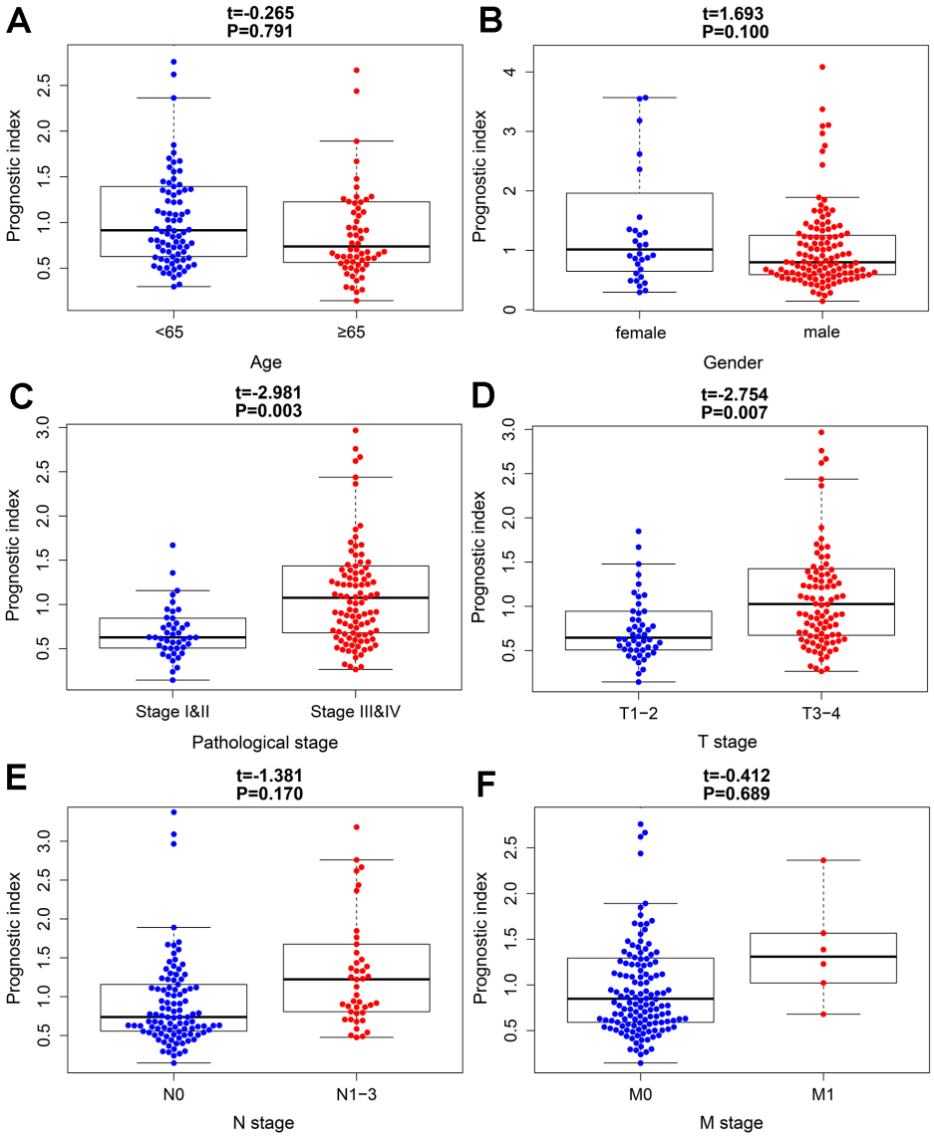
**The relationships between the immune-based prognostic index and clinicopathological factors.** (**A**) age; (**B**) gender; (**C**) pathological stage; (**D**) T stage; (**E**) N stage and (**F**) M stage in the high-risk (red) and low-risk (blue) groups of the BLCA.

**Figure 8:**
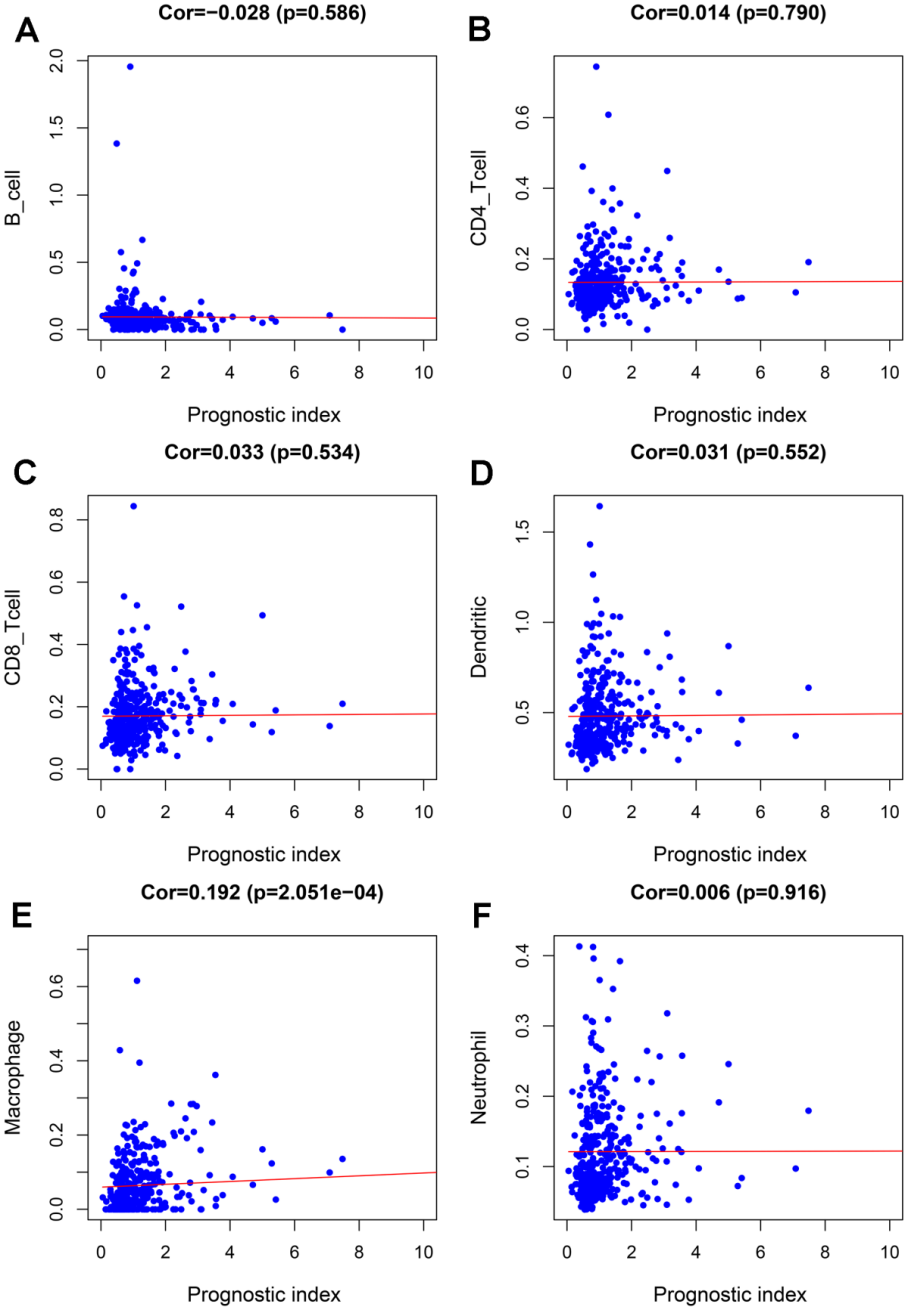
**Relationships between the abundances of six types of immune cells and the immune-based prognostic index in patients with BLCA.** (**A**) B cells; (**B**) CD4 T cells; (**C**) CD8 T cells; (**D**) dendritic cells; (**E**) macrophages; (**F**) neutrophils.

To further evaluate the clinical value of IRGPI, we analyzed the relationships between the nine survival-associated IRGs and IRGPI with clinicopathologic factors, including age, gender, pathological stage, T stage, N stage, and M stage (Table 3). IRGPI was an independent predictor. It showed statistically significant differences in terms of pathological and T-stage, but no statistically significant differences in terms of age, gender, N stage, or M stage (Figure 7). These findings suggest that IRGPI accurately predicts the pathological stages of BLCA. We also evaluated the relationships between the abundances of six types of immune cells and the immune-based prognostic index to determine whether the IRGPI accurately reflected the tumor immune microenvironment status. We found that IRGPI significantly correlated with macrophages (Figure 8E). There was no significant correlation between IRPGI and five types of immune cells, including B cells (Figure 8A), CD4+ T cells (Figure 8B), CD8+ T cells (Figure 8C), dendritic cells (Figure 8D), or neutrophils (Figure 8F).

**Table 3:** The relationship between the expression of the survival-associated IRGs and clinicopathological factors in BLCA.

**Genes**	**Age(>65/≤65)**			**Gender** **(male/female)**			**Pathological stage** **(IV-III/I-II)**			**T stage (T3-T4/T1-** **T2)**			**N stage (N1-3/N0)**			**M stage (M1/ M0)**	
	**t**	***P***		**t**	***P***		**t**	***P***		**t**	***P***		**t**	***P***		**t**	***P***
MMP9	-0.078	0.938		0.658	0.513		-1.636	0.105		-1.991	0.048		-0.566	0.573		-1.089	0.322
PDGFRA	0.538	0.591		0.849	0.401		-3.077	0.002		-3.040	0.003		-1.203	0.232		-0.462	0.660
AHNAK	0.760	0.449		1.447	0.157		-3.700	<0.001		-3.933	<0.001		-2.238	0.028		0.942	0.382
OAS1	-2.146	0.034		-2.543	0.013		2.159	0.034		2.148	0.034		1.775	0.079		2.929	0.023
OLR1	-0.763	0.448		0.024	0.981		-1.094	0.277		-1.059	0.292		-0.364	0.717		2.989	0.004
RAC3	0.382	0.703		1.040	0.305		-0.544	0.588		0.066	0.947		-1.703	0.093		-1.129	0.309
IGF1	-0.094	0.925		0.856	0.397		-3.557	<0.001		-3.111	0.002		-1.090	0.279		-0.965	0.373
PGF	0.343	0.732		-0.634	0.529		0.883	0.380		0.270	0.788		2.090	0.039		-0.851	0.431
SH3BP2	-0.798	0.427		0.214	0.832		1.799	0.077		0.900	0.371		2.546	0.012		-0.121	0.908
riskScore	-0.265	0.791		1.693	0.100		-2.981	0.003		-2.754	0.007		-1.381	0.170		-0.412	0.689

## DISCUSSION

Bladder cancer is a common malignant tumor of the urinary system and has become the 10th most common malignant tumor in Europe and America [1]. About 70% of bladder cancers are non-muscular invasive bladder cancer, and 30% are muscular invasive bladder cancer. Non-muscular invasive bladder cancer is characterized by a high recurrence rate and low mortality, while about 50% of muscular invasive bladder cancer is potentially lethal [24]. The clinical manifestations of non-muscular invasive bladder cancer are heterogeneous, and it is essential to accurately predict the risk of progression of non-muscular invasive bladder cancer. Accurate identification of high-risk populations and formulation of optimal treatment plans in a timely fashion are clinical problems that need to be resolved. Previous studies showed that the prognosis of patients undergoing cystectomy due to the progression of non-muscle invasive bladder cancer is worse than that of patients newly diagnosed with muscle-invasive bladder cancer directly undergoing cystectomy [25]. This suggests that early cystectomy improves outcomes in those at high risk for progression.

The immune system recognizes and eliminates tumor cells; however, tumor cells can evade the immune system through immune escape and immune suppression. Abnormal immune responses are closely related to the occurrence and progression of tumors [26]. Studies found that IRGs play critical regulatory roles in immune responses, including the following: regulation of differentiation and development of bone marrow hematopoietic stem cells; regulation of the development, differentiation, and activation of immune cells; and participation in the activation of autophagy and inflammatory processes [27]. Studies showed that IRGs predict survival and outcomes for various tumors, and they are potential targets for tumor therapy [5, 7, 8, 28, 29].

The development of high-throughput sequencing technology gave rise to the combination of microarray data and bioinformatics for tumor diagnosis and the discovery of prognostic biomarkers. Data mining technology generates gene signatures containing various relevant genes. These gene signatures are widely used in molecular diagnosis, individualized treatment, and survival prediction [30]. Their predictive accuracy is better than those of single biomarkers [31].

For these reasons, it is desirable to use bioinformatics technology to establish immune-related gene signatures to guide treatment and predict outcomes for patients with BLCA. We conducted a comprehensive analysis of the BLCA gene expression profile to identify IRGs that play central roles in the development and outcomes of BLCA. We identified nine IRGs (MMP9, PDGFRA, AHNAK, OAS1, OLR1, RAC3, IGF1, PGF, and SH3BP2) to predict OS using univariate and multivariate Cox proportional hazard regression models. We used the expression levels of these IRGs to establish a prediction model. This method is more economical and clinically feasible than whole-genome sequencing. The combination of nine gene signatures with clinicopathological parameters can enable clinicians to determine individual outcomes more accurately. The risk scoring system is easy to understand and helps customize treatment plans. The ROC curve, Kaplan–Meier analysis, and internal verification showed that this model accurately predicted the OS of BLCA. The correlation analysis between clinicopathological and risk scores showed that the risk scores were related to the pathological and T-stage.

We also explored the ability of risk score and other clinicopathological parameters to predict survival and found that risk score was an independent prognostic indicator of BLCA.

Some of the nine IRGs participate in the development of BLCA and affect outcomes, and some have not been reported. A study reported that LINC01605 up-regulated the expression of MMP9 to promote proliferation, migration, and invasion of BLCA cells [32]. PDGFRA is up-regulated in BLCA tissues, which is significantly related to tumor prognosis and can be used as a prognostic marker of BLCA [33]. In urine cytology, BLCA can be distinguished from benign urothelial lesions by detecting ANHAK [34]. A study found that ANHAK was significantly related to the outcomes of BLCA [35]. OAS1 was significantly related to outcomes of BLCA and can be used as a prognostic marker [35]. RAC3 is highly expressed in bladder cancer tissues and can promote the proliferation, migration, and invasion of bladder cancer cells [36]. A study reported that plasma IGF1 is highly expressed in patients with bladder cancer; measuring plasma IGF1 values can help assess bladder cancer risk [37]. There is no report on the roles of OLR1, PGF, or SH3BP2 in outcomes of bladder cancer.

We also focused on the relationship between risk score and tumor microenvironment to reveal its potential clinical significance. The risk score reflects the infiltration state of macrophages. The higher the risk score, the higher the abundance of macrophages, suggesting that higher amounts of abnormal expression of immune genes correspond to a higher abundance of macrophages in the tumor immune microenvironment; this, in turn, participates in the occurrence and progression of BLCA and the processes of invasion and metastasis. Tumor-associated macrophages are part of the tumor microenvironmental cells and affect the progress of solid tumors. Studies have found that macrophages can directly affect the immune response to bladder cancer induced by Bacillus Calmette-Guerin [38]. In addition, studies have found that exosomes miR-21 can promote cancer progression through polarized tumor-associated macrophages [39].

This study may provide new insights into the molecular mechanisms, immunotherapy, and prognostic predictions of BLCA. One of the advantages of the BLCA predictive risk-scoring model constructed in this study is its high sensitivity and specificity for predicting OS. Random internal verification demonstrated its effectiveness. The risk scoring model is related to the immunosuppressive environment and immune checkpoint expression and may help clinicians plan personalized immunotherapy for patients with BLCA.

This study also has some limitations. First, the risk scoring model needs to be further validated in multi-center clinical trials and prospective studies. Second, further research on the functions and mechanisms of the nine IRGs is needed.

### Data availability

All data generated or analyzed during this study are included in this article.
